# Osteoblastic glucocorticoid signaling exacerbates high-fat-diet- induced bone loss and obesity

**DOI:** 10.1038/s41413-021-00159-9

**Published:** 2021-09-01

**Authors:** Sarah Kim, Holger Henneicke, Lauryn L. Cavanagh, Eugenie Macfarlane, Lee Joanne Thai, Daphne Foong, Sylvia J. Gasparini, Colette Fong-Yee, Michael M. Swarbrick, Markus J. Seibel, Hong Zhou

**Affiliations:** 1 Bone Research Program, ANZAC Research Institute, The University of Sydney, Sydney, NSW Australia; 2 Concord Clinical School, The University of Sydney, Sydney, NSW Australia; 3 Department of Medicine III, Technische University Dresden Medical Center, Dresden, Germany; 4 Center for Healthy Aging, Technische Universität Dresden Medical Center, Dresden, Germany; 5 Center for Regenerative Therapies Dresden, Technische University Dresden, Dresden, Germany; 6 Department of Endocrinology and Metabolism, Concord Repatriation General Hospital, The University of Sydney, Sydney, NSW Australia

**Keywords:** Osteoporosis, Pre-diabetes, Bone, Obesity

## Abstract

Chronic high-fat diet (HFD) consumption not only promotes obesity and insulin resistance, but also causes bone loss through mechanisms that are not well understood. Here, we fed wild-type CD-1 mice either chow or a HFD (43% of energy from fat) for 18 weeks; HFD-fed mice exhibited decreased trabecular volume (−28%) and cortical thickness (−14%) compared to chow-fed mice. In HFD-fed mice, bone loss was due to reduced bone formation and mineral apposition, without obvious effects on bone resorption. HFD feeding also increased skeletal expression of sclerostin and caused deterioration of the osteocyte lacunocanalicular network (LCN). In mice fed HFD, skeletal glucocorticoid signaling was activated relative to chow-fed mice, independent of serum corticosterone concentrations. We therefore examined whether skeletal glucocorticoid signaling was necessary for HFD-induced bone loss, using transgenic mice lacking glucocorticoid signaling in osteoblasts and osteocytes (HSD2^OB/OCY^-tg mice). In HSD2^OB/OCY^-tg mice, bone formation and mineral apposition rates were not suppressed by HFD, and bone loss was significantly attenuated. Interestingly, in HSD2^OB/OCY^-tg mice fed HFD, both Wnt signaling (less sclerostin induction, increased β-catenin expression) and glucose uptake were significantly increased, relative to diet- and genotype-matched controls. The osteocyte LCN remained intact in HFD-fed HSD2^OB/OCY^-tg mice. When fed a HFD, HSD2^OB/OCY^-tg mice also increased their energy expenditure and were protected against obesity, insulin resistance, and dyslipidemia. Therefore, glucocorticoid signaling in osteoblasts and osteocytes contributes to the suppression of bone formation in HFD-fed mice. Skeletal glucocorticoid signaling is also an important determinant of glucose uptake in bone, which influences the whole-body metabolic response to HFD.

## Introduction

In mice, chronic consumption of high-fat diets (HFDs), particularly those high in saturated fat, not only leads to obesity,^1^ insulin resistance,^2^ and dyslipidemia^3^ but also produces a marked deterioration in bone structure, mass, and strength.^4–11^ Similarly, studies in humans have shown that higher dietary saturated fat intake is associated with lower bone mineral density and increased fracture risk in both men and women.^12,13^

Bone loss in mice fed HFDs (deriving up to 60% of energy from fat) is thought to result from either excessive bone resorption,^4,6^ reduced bone formation,^10,11^ or a combination of the two,^8^ with the effects generally being more pronounced in younger mice.^14,15^

Glucocorticoids are steroid hormones that mediate the physiological response to stress^16^ and have deleterious effects on bone structure and quality. In humans, long-term use of exogenous glucocorticoids inhibits bone formation, leading to bone loss and ultimately osteoporosis.^17,18^ Similarly, in mice, chronic mild stress increases serum corticosterone concentrations and activates skeletal glucocorticoid signaling, leading to bone loss.^19^

Although circulating glucocorticoid levels are controlled by the hypothalamic–pituitary–adrenal axis, glucocorticoid actions in cells and tissues depend on the activities of the enzymes 11β-hydroxy-steroid dehydrogenase type 1 (11β-HSD1) and type 2 (11β-HSD2). 11β-HSD1 converts inactive cortisone (11-dehydrocorticosterone in rodents) to active cortisol (corticosterone),^20^ whereas 11β-HSD2 catalyzes the opposite reaction, inactivating intracellular glucocorticoids at the prereceptor level. Regulation of intracellular glucocorticoid activity is critical for the metabolic response to HFD, as mice lacking 11β-HSD1 are resistant to visceral fat accumulation and glucose intolerance when fed a HFD.^21^

In the present study, we sought to investigate the mechanisms underlying HFD-induced bone loss in mice and to investigate whether endogenous glucocorticoid signaling is required for the skeletal and metabolic responses to a HFD.

## Results

### HFD feeding increased local glucocorticoid signaling in bone

Here, we studied male mice with disrupted glucocorticoid signaling in mature osteoblasts and osteocytes (HSD2^OB/OCY^-tg mice) and their wild-type littermates, both on a CD-1 background. HSD2^OB/OCY^-tg mice expressed the rat 11β-HSD2 transgene in osteoblasts and osteocytes under the control of the Col2.3 promoter.^22,23^ Skeletal expression of 11β-HSD2 protein was confirmed by RT-PCR and immunohistochemistry.^24^ From 7 weeks of age, mice were fed either regular chow or HFD (43% of calories from fat) for 8 and 18 weeks.

**Fig. 1:**
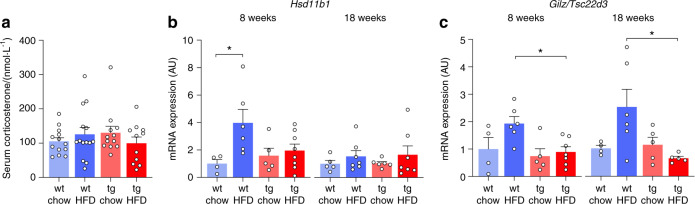
HFD feeding induced skeletal glucocorticoid signaling in wild-type but not HSD2^OB/OCY^-tg mice, independent of circulating corticosterone concentrations. The results are shown as scatterplots, with the mean ± SEM shown in columns. **a** Corticosterone concentrations were measured in serum collected from mice between 10 a.m. and 2 p.m. wt wild-type, tg transgenic. **b** Skeletal (femur) mRNA expression of the 11β-hydroxy-steroid-dehydrogenase type 1 (11β-HSD1) gene (*Hsd11b1*) after 8 and 18 weeks of chow or HFD feeding. Gene expression results are shown in arbitrary units (AU) relative to wild-type chow-fed mice (1.00). At 8 weeks, there was a significant effect of diet (*F*_1,19_ = 5.932, *P* = 0.025). **c** Skeletal (femur) mRNA expression of the glucocorticoid target gene *Tsc22 domain family member 3* (*Tsc22d3*, also known as *glucocorticoid-induced leucine zipper* (*Gilz*)). At 8 weeks, the effect of genotype was significant (*F*_1,18_ = 5.622, *P* = 0.029), while there was a significant diet × genotype interaction at 18 weeks (*F*_1,16_ = 5.556, *P* = 0.032). Significance was determined using two-way ANOVA at each timepoint, with the Tukey’s multiple comparison test used for post hoc testing. In all cases, *P < 0.05 for comparison

First, we assessed both systemic glucocorticoid levels (serum corticosterone concentrations) and skeletal glucocorticoid activation (mRNA expression of *Hsd11b1*, the gene encoding 11β-HSD1, and the glucocorticoid target gene *glucocorticoid-induced leucine zipper* (*Gilz*)^25^). Serum corticosterone concentrations did not differ between wild-type and transgenic mice, and they were not altered by HFD feeding (Fig. 1a). HFD feeding induced skeletal *Hsd11b1* mRNA expression in wild-type mice but not in HSD2^OB/OCY^-tg mice (Fig. 1b). *Gilz/Tsc22d3* mRNA was also induced by HFD feeding in wild-type mice, but there was no effect in HSD2^OB/OCY^-tg mice (Fig. 1c). Therefore, chronic HFD feeding induced skeletal glucocorticoid signaling in wild-type mice but not HSD2^OB/OCY^-tg mice, and this induction did not depend on systemic glucocorticoid concentrations.

### Disrupting glucocorticoid signaling in osteoblasts and osteocytes prevented HFD-induced bone loss

After 8 weeks of dietary intervention, there were no significant effects of HFD on bone volume (BV/TV) or cortical thickness (Ct.Th) in male wild-type or HSD2^OB/OCY^-tg mice (Fig. S1). At this timepoint, the effects of diet on bone parameters were subtle: a statistically significant reduction in trabecular spacing was observed in HFD-fed HSD2^OB/OCY^-tg mice but not in wild-type mice (*P* = 0.028, Fig. S1c). Instead, the effects of genotype predominated, with HSD2^OB/OCY^-tg mice displaying significantly increased trabecular thickness (Tb.Th.), increased trabecular spacing, reduced trabecular number (Tb.N.), and increased Ct.Th compared to wild-type mice. These effects were consistent with our previous studies.^19,24,26^ Female HSD2^OB/OCY^-tg mice fed HFD displayed a similar bone phenotype but were not studied further, as they did not develop insulin resistance (data not shown).

**Fig. 2:**
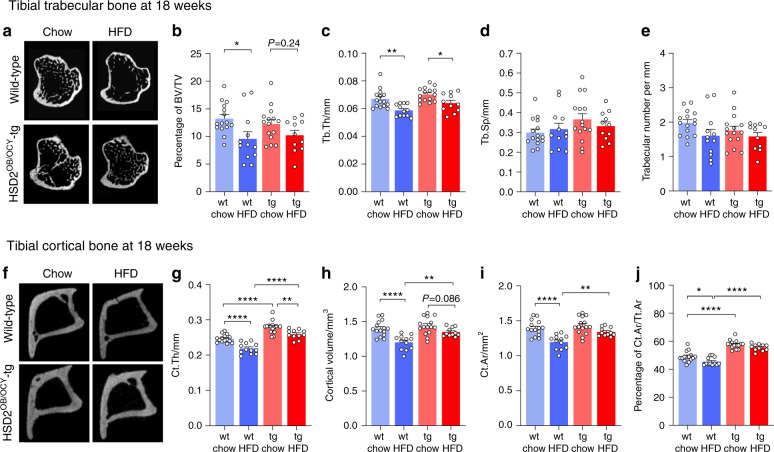
Disrupting glucocorticoid signaling in osteoblasts and osteocytes prevented HFD-induced loss of trabecular and cortical bone. **a** Sections of tibial trabecular bone from male wild-type (wt) and HSD2^OB/OCY^-tg (tg) mice fed either chow or HFD for 18 weeks. **b**–**e** Micro-CT results are shown as scatterplots, with the mean ± SEM shown in columns, *n* = 11–15/group, as shown. **b** Bone volume/total volume (BV/TV) in trabecular bone; the effect of diet was highly significant (*F*_1,49_ = 9.511, *P* = 0.003 4). **c** Trabecular thickness (Tb.Th.); the effect of diet was highly significant (*F*_1,49_ = 20.50, *P* < 0.000 1), and the effect of genotype was significant (*F*_1,49_ = 7.010, *P* = 0.011). **d** Trabecular spacing (Tb.Sp.). **e** Trabecular number (Tb.N.); the effect of diet was significant (*F*_1,49_ = 4.367, *P*  = 0.042). **f** Sections of tibial cortical bone from male wild-type (wt) and HSD2^OB/OCY^-tg (tg) mice fed either chow or HFD for 18 weeks. **g–j** Cortical micro-CT measurements of *n* = 11–15/group, as shown: **g** Cortical thickness (Ct.Th); the effects of diet and genotype were both highly significant (diet: *F*_1,49_ = 36.15, *P*  < 0.000 1, genotype: *F*_1,49_ = 83.45, *P*  < 0.000 1). **h** Cortical volume; the effects of diet and genotype were highly significant (diet: *F*_1,49_ = 23.43, *P* < 0.000 1, genotype: *F*_1,49_ = 10.03, *P*  = 0.002 7). **i** Cortical area (Ct.Ar); the effects of diet and genotype were highly significant (diet: *F*_1,49_ = 24.34, *P*  < 0.000 1, genotype: *F*_1,49_ = 9.878, *P*  = 0.002 8). **j** Cortical area fraction (Ct.Ar/Tt.Ar); the effects of diet and genotype were highly significant (diet: *F*_1,49_ = 11.16, *P* = 0.001 6, genotype: *F*_1,49_ = 119.9, *P*  < 0.000 1). Significance was determined using two-way ANOVA, with the Tukey’s multiple comparison test used for post hoc testing. In all cases, *P  < 0.05, **P  < 0.01, ***P  < 0.001, and ****P  < 0.000 1 for comparison

After 18 weeks, however, the effects of the HFD were well established, and overall, they were much greater in wild-type mice than in HSD2^OB/OCY^-tg mice (Fig. 2). In wild-type mice, 18 weeks of HFD feeding significantly reduced trabecular bone volume (by 28%, *P* < 0.05 compared to chow, Fig. 2b); this effect was attenuated in HSD2^OB/OCY^-tg mice, where trabecular bone volume was reduced by 17% (*P* = 0.24 vs. chow). HFD feeding also significantly reduced Tb.Th. in both wild-type and transgenic mice, by 12% (*P* < 0.01) and 9% (*P* < 0.05) respectively, compared to chow-fed controls (Fig. 2c). Tb.N. was also reduced after 18 weeks of HFD feeding, by 19% in wild-type mice and by 10% in HSD2^OB/OCY^-tg mice (overall effect of diet: *P* = 0.042, Fig. 2e).

HFD feeding also led to the loss of cortical bone: the effects were generally smaller in magnitude than those observed in trabecular bone and were significantly attenuated in HSD2^OB/OCY^-tg mice. Specifically, 18 weeks of HFD feeding led to significant reductions in Ct.Th in both wild-type (11% reduction, *P* < 0.000 1 vs. chow) and HSD2^OB/OCY^-tg (7% reduction, *P* < 0.01) mice compared to chow-fed controls (Fig. 2g). HFD feeding also reduced cortical bone volume and area by 14% (*P* < 0.000 1) in wild-type mice but by only ~6% in HSD2^OB/OCY^-tg mice (*P* > 0.05 for each, Fig. 2h, i). In each case, the effects of genotype were also highly significant (*P* < 0.01). HFD feeding for 18 weeks also reduced the Ct.Ar fraction in wild-type mice (7% decrease, *P* < 0.05 vs. chow), but no significant change was observed in HSD2^OB/OCY^-tg mice (4% decrease, *P* = ns, Fig. 2j).

In summary, 18 weeks of HFD feeding led to significant losses of both trabecular and cortical bone volumes in wild-type mice. In contrast, the deleterious effects of HFD feeding were clearly attenuated in mice lacking glucocorticoid signaling in osteoblasts and osteocytes.

### HFD feeding inhibited bone formation in wild-type but not HSD2^OB/OCY^-tg mice

**Fig. 3:**
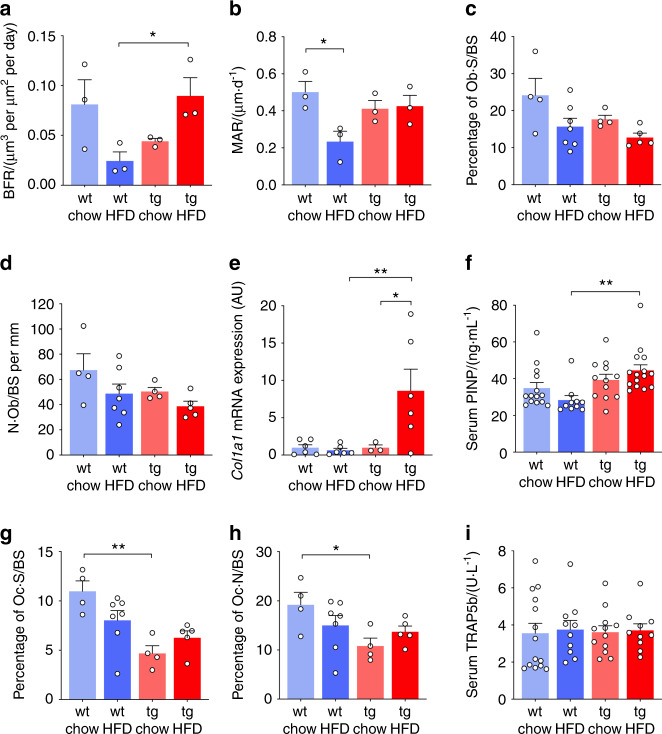
HFD feeding for 18 weeks reduced bone formation in wild-type mice but not in HSD2^OB/OCY^-tg mice. The results are shown as scatterplots, with the mean ± SEM shown in columns. **a** Bone formation rate (BFR) and **b** mineral apposition rate (MAR) in wild-type (wt) and HSD2^OB/OCY^-tg (tg) mice fed either chow or HFD for 18 weeks. In each case, there was a significant diet × genotype interaction (for BFR, *F*_1,8_ = 10.16, *P* = 0.013; for MAR: *F*_1,8_ = 6.68, *P* = 0.032). **c** Trabecular osteoblast surface (%): the effect of diet was significant (*F*_1,16_ = 6.74, *P* = 0.020). **d** Osteoblast number. **e** Bone mRNA expression of *collagen, type 1, alpha 1* (*Col1a1*): the diet × genotype interaction was significant (*F*_1,17_ = 5.010, *P* = 0.039). **f** Serum procollagen type I N-terminal propeptide (P1NP) concentrations: the effect of genotype was highly significant (*F*_1,47_ = 11.47, *P* = 0.001 4). **g** Osteoclast surface: the diet × genotype interaction was significant (*F*_1,16_ = 5.400, *P* = 0.034). **h** Osteoclast number: the effect of genotype was significant (*F*_1,16_ = 5.932, *P* = 0.027). **i** Serum tartrate-resistant acid phosphatase 5b (TRAP5b) concentrations. In all cases, significance was determined using two-way ANOVA, with the Tukey’s multiple comparison test used for post hoc testing, *P < 0.05 and **P < 0.01 for comparison

Dynamic histomorphometry analysis at the 18-week timepoint revealed that the effects of HFD on the bone-formation rate (BFR) were different between wild-type and transgenic mice (diet × genotype interaction: *P* = 0.013, Fig. 3a). Similarly, HFD feeding reduced the mineral apposition rate (MAR) in wild-type mice (by 54%, *P* < 0.05 vs. chow), while in HSD2^OB/OCY^-tg mice, the MAR was instead maintained upon HFD feeding. Overall, the effects of HFD on the MAR were significantly different between wild-type and HSD2^OB/OCY^-tg mice (diet × genotype interaction: *P* = 0.032, Fig. 3b).

HFD feeding had a significant effect on the trabecular osteoblast surface (effect of diet: *P* = 0.020, Fig. 3c), but it did not significantly reduce the osteoblast number (Fig. 3d). There were no significant effects of genotype on the osteoblast surface or osteoblast number. Therefore, bone formation was negatively affected by HFD feeding in wild-type mice but not in mice with disrupted glucocorticoid signaling in osteoblasts and osteocytes.

Since the above changes occurred without significant alterations in osteoblast number, we next investigated whether a HFD may instead affect osteoblast activity. Skeletal *Col1a1* mRNA expression was significantly increased by HFD feeding in HSD2^OB/OCY^-tg mice (8.6-fold vs. chow), but this did not occur in wild-type mice (Fig. 3e). Consistent with increased osteoblast activity, serum procollagen type I N-terminal propeptide (PINP) levels were significantly increased in HSD2^OB/OCY^-tg mice after 18 weeks of HFD feeding, indicative of increased bone formation (Fig. 3f).

Changes in bone mass also depend on changes in bone resorption. Both the osteoclast surface (effect of genotype: *P* = 0.000 8, Fig. 3g) and osteoclast number (effect of genotype: *P* = 0.027, Fig. 3h) were significantly lower in HSD2^OB/OCY^-tg mice than in wild-type mice. The effects of diet on these parameters were not significant, however. Serum concentrations of TRAB5b, a marker of osteoclast activity, were not affected by either diet or genotype (Fig. 3i).

### HFD feeding enhances skeletal Wnt signaling in HSD2^OB/OCY^-tg mice

**Fig. 4:**
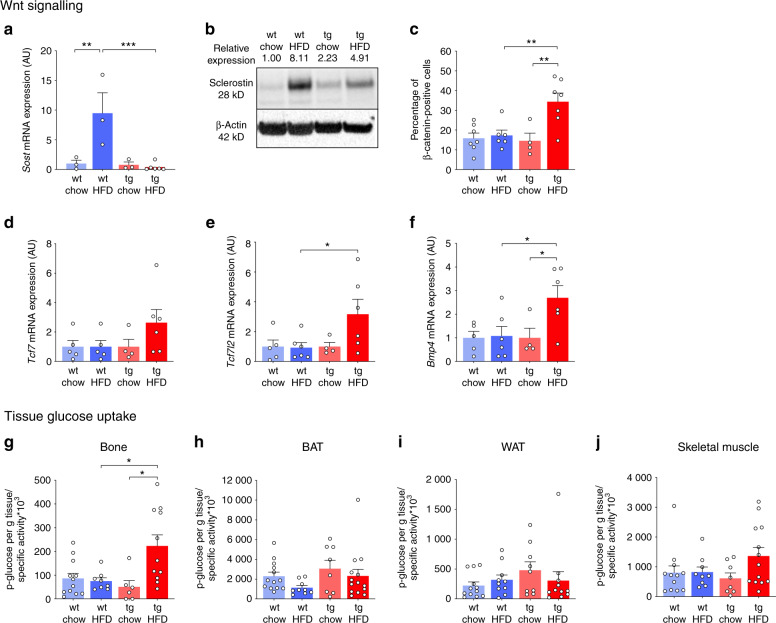
Wnt signaling and glucose uptake in the bones of wild-type and HSD2^OB/OCY^-tg mice. The results are shown as scatterplots, with the mean ± SEM shown in columns. **a** Bone expression of sclerostin (*Sost*) mRNA. All mRNA expression results are shown relative to chow-fed wild-type mice and are expressed in arbitrary units (AU), normalized to *18S* mRNA expression. The diet × genotype interaction was highly significant (*F*_1,11_ = 9.71, *P* = 0.009 8). **b** Immunoblot of sclerostin protein expression in bones of wild-type and HSD2^OB/OCY^-tg mice: each lane contains samples pooled from three mice, with β-actin shown as a loading control. **c** Proportion of β-catenin-positive osteoblasts and osteocytes. The diet × genotype interaction was significant (*F*_1,20_ = 6.488, *P* = 0.019). Representative sections of trabecular bone from each group of mice are shown in Fig. S2. **d**–**f** Bone mRNA expression of the Wnt signaling molecules **d** *transcription factor 7 (T-cell specific, HMG-box)* (*Tcf7*), **e** *transcription factor 7 like 2* (*Tcf7l2*), and **f** *bone morphogenetic protein-4* (*Bmp4*). **g** Glucose uptake into bone; **h** brown adipose tissue; **i** gonadal white adipose tissue; and **j** quadriceps muscle after 8 weeks of chow/FHD deeding. For glucose uptake measurements, *n* = 6–14 for each group. For glucose uptake in bone, the effect of genotype was significant (*F*_1,33_ = 4.729, *P* = 0.037). Significance was determined using two-way ANOVA, with the Tukey’s multiple comparison test used for post hoc testing. In all cases, *P < 0.05, **P < 0.01, and ***P < 0.001 for comparison

Wnt signaling is critical for bone formation^27^ and may be influenced by HFD feeding.^10^ We therefore investigated skeletal Wnt signaling pathways in wild-type and transgenic mice fed either chow or HFD by measuring mRNA and protein expression levels of *Sost*/sclerostin, β-catenin staining in sections of trabecular bone, and mRNA expression of the nuclear effectors of canonical Wnt signaling *Tcf7/Tcf1* and *Tcf7l2*/*Tcf4* and the osteogenic factor *Bmp4*. In wild-type mice, skeletal *Sost* mRNA expression was increased 8.3-fold by HFD feeding but remained unchanged in HFD-fed HSD2^OB/OCY^-tg mice (diet × genotype interaction: *P* = 0.0098, Fig. 4a). Similarly, sclerostin protein expression was induced by HFD greater than eightfold in wild-type mice, but this increase was attenuated in HSD2^OB/OCY^-tg mice (Fig. 4b). The proportion of β-catenin-positive osteoblasts and osteocytes was approximately doubled in sections from HFD-fed HSD2^OB/OCY^-tg mice compared to the other groups (diet × genotype interaction: *F*_1,20_ = 6.488, *P* = 0.019, Fig. 4c and S2). Skeletal expression of *Tcf7*, *Tcf7l2*, and *Bmp4* was increased in HFD-fed HSD2^OB/OCY^-tg mice compared to chow-fed mice (by 2.64-, 3.17-, and 2.70-fold, Fig. 4d–f). However, in wild-type mice, skeletal expression of these transcription factors and signaling molecules was not induced by HFD feeding. Therefore, in wild-type mice, HFD feeding reduced Wnt signaling by increasing the expression of *Sost*/sclerostin, while in HSD2^OB/OCY^-tg mice, Wnt signaling was augmented by HFD feeding, as evidenced by an increased proportion of β-catenin-positive cells and increased expression of downstream signaling molecules.

### Skeletal glucose uptake increases in HSD2^OB/OCY^-tg mice in response to HFD feeding

In vivo, bone formation is energetically expensive, requiring an upregulation of glucose uptake and metabolism in osteoblasts.^28–34^ Wnt signaling promotes bone formation by stimulating glucose metabolism in osteoblasts.^30,35^ Given the increased bone formation observed in HFD-fed HSD2^OB/OCY^-tg mice and the requirement for Wnt signaling, we measured skeletal glucose uptake after 8 weeks of chow/HFD feeding.

In HSD2^OB/OCY^-tg mice, but not wild-type mice, glucose uptake into bone was increased 4.5-fold in response to HFD feeding (*P* < 0.05, Fig. 4g). Increased glucose uptake in bone was not attributable to differences in the mRNA expression of the glucose transporters *Glut1* and *Glut4* (Fig. S3). Interestingly, glucose uptake into other insulin-sensitive tissues (brown and gonadal white adipose tissue, quadriceps muscle) was not altered (Fig. 4h–j). Therefore, the HFD-induced increase in bone formation seen in HSD2^OB/OCY^-tg mice was supported by a marked increase in skeletal glucose uptake.

### HSD2^OB/OCY^-tg mice were protected against HFD-induced loss of the lacunocanalicular network (LCN)

**Fig. 5:**
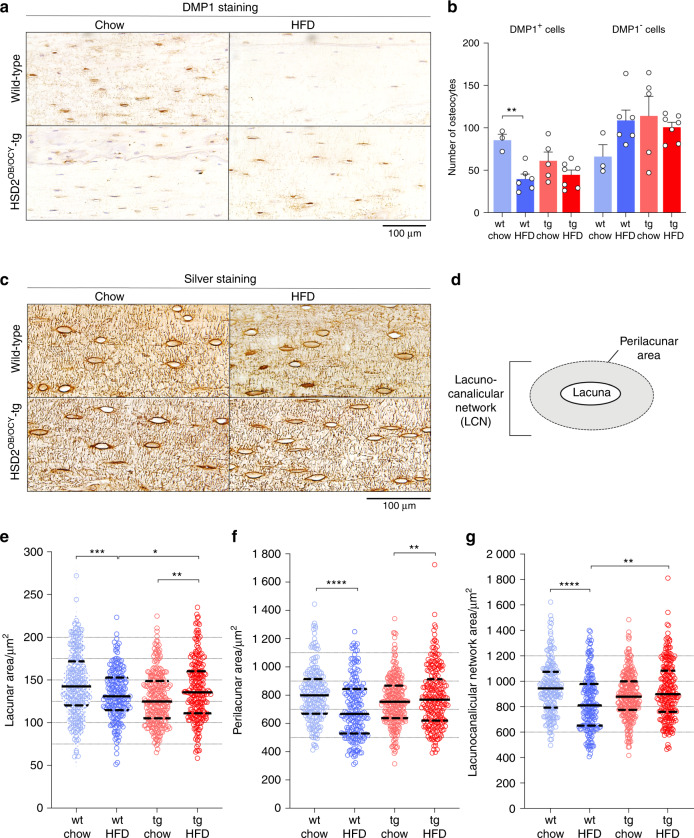
HFD feeding disrupted the lacunocanalicular network (LCN) in wild-type mice but not in HSD2^OB/OCY^-tg mice. **a** Representative images of DMP1-stained tibial sections from wild-type and HSD2^OB/OCY^-tg mice fed either chow or HFD for 18 weeks. **b** Number of DMP1-positive and DMP1-negative osteocytes in sections from wild-type and HSD2^OB/OCY^-tg mice. The results are shown as scatterplots, with the mean ± SEM shown in columns. Significance was determined using two-way ANOVA for DMP1^+^ and DMP1^−^ cells, with the Tukey’s multiple comparison test used for post hoc testing. **c** Representative images of silver nitrate-stained tibial sections from wild-type and HSD2^OB/OCY^-tg mice fed either chow or HFD for 18 weeks. **d** Diagram for determination of the lacunar area, perilacunar area, and the entire LCN. **e** Distribution of the lacunar area; median and quartlies (1st and 3rd) are shown by solid and dashed lines, respectively. Dotted lines show bin sizes used for the *χ*^2^-test, which was used to compare distributions. Significance was adjusted for multiple comparisons using Bonferroni’s method. **f** Distribution of the perilacunar area. **g** Distribution of the entire LCN area. In all cases, *P < 0.05, **P < 0.01, ***P < 0.001, and ****P < 0.000 1 for comparisons

The 11β-HSD2 transgene is expressed in both osteocytes and osteoblasts in HSD2^OB/OCY^-tg mice. Since the majority (90%–95%) of cells in bone are osteocytes, we stained tibial sections from chow- and HFD-fed wild-type and HSD2^OB/OCY^-tg mice for expression of the osteocyte marker dentin matrix protein-1 (DMP1) (Fig. 5a). In wild-type mice, HFD feeding led to a 53% reduction in the number of DMP1-positive osteocytes (*P* < 0.01, Fig. 5b, left graph), but this effect was not observed in HSD2^OB/OCY^-tg mice. Diet did not affect the number of DMP1-negative osteocytes in either wild-type or transgenic mice (Fig. 5b, right graph). Closer examination of DMP1-stained osteocytes also indicated that the number of DMP1-positive osteocyte dendrites was reduced in wild-type, but not HSD2^OB/OCY^-tg mice, when fed HFD (Fig. 5a).

Next, we examined the potential for HFD-induced deterioration of the LCN.^36^ Analysis of the LCN and its components in silver-stained sections of tibiae from the above mice (Fig. 5c, d) indicated that HFD feeding produced differential effects between wild-type and HSD2^OB/OCY^-tg mice (Fig. 5e–g). In wild-type mice, HFD led to a marked shift toward smaller lacunae (8% reduction in median lacunar size vs. chow, *P* < 0.001), but this was not observed in transgenic mice, where a shift toward larger lacunae was observed instead (9% increase in median lacunar size, *P* < 0.01 vs. chow, Fig. 5e). Similar changes were observed for the perilacunar area (Fig. 5f) and for the entire LCN (Fig. 5g). Overall, HDS2^OB/OCY^-tg mice were protected against the HFD-induced disruption of the LCN observed in wild-type mice.

### HSD2^OB/OCY^-tg mice were protected against HFD-induced insulin resistance and glucose intolerance

**Fig. 6:**
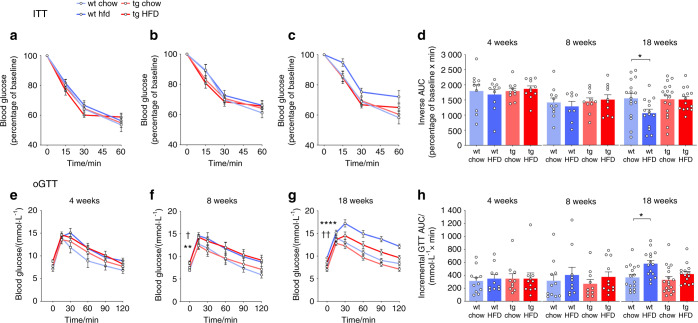
HSD2^OB/OCY^-tg mice were protected against HFD-induced insulin resistance and glucose intolerance. **a–d** Insulin tolerance test (ITT) results from wild-type chow, wild-type HFD, tg chow and tg HFD mice after 4, 8, and 18 weeks of feeding. Blood glucose concentrations are expressed as a percentage of basal levels and are shown as the mean ± SEM, *n* = 10–11/group. **d** Inverse area under the curve during the ITT at 4, 8, and 18 weeks. The results are shown as scatterplots, with the mean ± SEM shown in columns. Significance was determined using two-way ANOVA at each timepoint, with the Tukey’s multiple comparison test used for post hoc testing. *P < 0.05 for comparison. **e–h** Oral glucose tolerance test (oGTT) results after 4, 8, and 18 weeks of feeding, *n* = 14–17 for each group/timepoint. **h** Incremental area under the curve for the GTT at 4, 8, and 18 weeks. At 18 weeks, the effects of diet and genotype were both significant (diet: *F*_1,58_ = 9.640, *P* = 0.002 9; genotype: *F*_1,58_ = 4.139, *P* = 0.047). *P < 0.05, **P < 0.01, and ****P < 0.000 1 for wild-type chow vs. wild-type HFD, †*P* < 0.05 and ††*P* < 0.01 for tg chow vs. tg HFD

Chronic HFD consumption also impairs insulin action due to lipotoxicity.^37^ After 8 weeks, and more so after 18 weeks of HFD feeding, both wild-type and transgenic mice developed fasting hyperglycemia (Fig. 6). When fed a HFD, insulin resistance developed in wild-type mice but not in HSD2^OB/OCY^-tg mice (Fig. 6d). Fasting insulinemia was not affected by diet or genotype (Fig. S3). Wild-type mice, but not HSD2^OB/OCY^-tg mice, also developed impaired glucose tolerance after 18 weeks of HFD feeding (Fig. 6g, h).

### HSD2^OB/OCY^-tg mice were protected against HFD-induced obesity and dyslipidemia

**Fig. 7:**
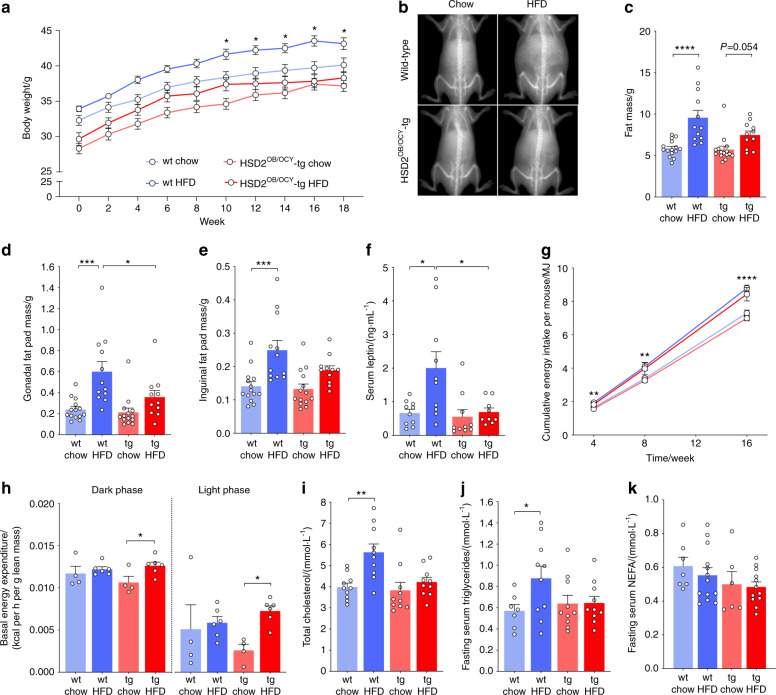
When fed a HFD, HSD2^OB/OCY^-tg mice gained less weight, due to increased energy expenditure, and were protected against HFD-induced dyslipidemia. **a** Body weight of wild-type and HSD2^OB/OCY^-tg mice fed either regular chow or a HFD ad libitum for 18 weeks. The results are shown as the mean ± SEM, *n* = 18, 15, 14, and 13 mice for wt chow, wt HFD, HSD2^OB/OCY^-tg chow, and HSD2^OB/OCY^-tg HFD, respectively. **b** Representative DEXA scans of wild-type and HSD2^OB/OCY^-tg mice after 18 weeks of either chow or HFD feeding. **c** Body composition of wild-type and HSD2^OB/OCY^-tg mice fed either chow or HFD for 18 weeks. The results are shown as scatterplots, with the mean ± SEM shown in columns. The effects of diet and genotype were significant (diet: *F*_1,49_ = 25.90, *P* < 0.000 1; genotype: *F*_1,49_ = 4.266, *P* = 0.044). **d** Gonadal fat pad mass at sacrifice. The effects of diet and genotype were significant (diet: *F*_1,47_ = 18.18, *P* < 0.000 1; genotype: *F*_1,47_ = 5.194, *P* = 0.027 2). **e** Inguinal fat pad mass at sacrifice. The effect of diet was highly significant (*F*_1,47_ = 19.53, *P* < 0.000 1). **f** Plasma leptin concentrations at sacrifice. **g** Cumulative food intake for chow- and HFD-fed wild-type and HSD2^OB/OCY^-tg mice. At each timepoint, *n* = 3–10 cages. **h** Energy expenditure in the dark (left) and light (right) phases, measured after 8 weeks of chow/HFD feeding. Energy expenditure was normalized to lean body mass. In the dark phase, the overall effect of diet was significant (*F*_1,16_ = 5.146, *P* = 0.038). **i** Fasting serum concentrations of cholesterol. The effects of diet and genotype were significant (diet: *F*_1,36_ = 10.78, *P* = 0.002 3; genotype: *F*_1,36_ = 6.268, *P* = 0.017). **j** Fasting serum concentrations of triglycerides and **k** nonesterified fatty acids (NEFAs). Significance was determined using two-way ANOVA, with Tukey’s multiple comparison tests used for post hoc testing. In all cases, *P < 0.05, **P < 0.01, ***P < 0.001, and ****P < 0.000 1 for the indicated comparison

We also analyzed the changes in the body weight and body composition of wild-type and HSD2^OB/OCY^-tg mice in response to HFD feeding. As in our previous studies,^19,26^ the initial body weight was ~4 g lower in HSD2^OB/OCY^-tg mice than in wild-type mice (effect of genotype, *F*_1,56_ = 34.46, *P* < 0.000 1, Fig. 7a). Regardless of their starting weights, the effects of HFD feeding on body weight and adiposity were attenuated in HSD2^OB/OCY^-tg mice compared to wild-type mice: 18 weeks of HFD feeding increased fat mass in wild-type mice by 63% (*P* < 0.000 1 vs. chow) but only by 30% in transgenic mice (*P* = 0.054 vs. chow, Fig. 7c). In wild-type mice, fat accumulation occurred mostly in gonadal (central) fat, which was increased 2.5-fold in mass by HFD feeding (*P* < 0.000 1 vs. chow, Fig. 7d). In HSD2^OB/OCY^-tg mice, however, HFD feeding did not significantly increase gonadal fat mass (68% increase, *P* = 0.44 vs. chow). Consistent with the differences in body fat accumulation, HFD feeding was accompanied by increased serum leptin concentrations in wild-type but not transgenic mice (diet × genotype interaction: *P* = 0.042, Fig. 7e).

To ascertain the cause of the differences in adiposity, we measured food intake and energy expenditure. In both wild-type and transgenic mice, cumulative caloric intake was significantly increased upon HFD feeding but was not different between genotypes (Fig. 7f). HFD feeding led to a significant increase in energy expenditure in HSD2^OB/OCY^-tg mice (*P* < 0.05 for each phase, Fig. 7h), while it remained unchanged in wild-type mice. Taken together, these results indicated that selectively disrupting glucocorticoid signaling in osteoblasts and osteocytes prevented HFD-induced central obesity by increasing energy expenditure.

In wild-type mice but not HSD2^OB/OCY^-tg mice, HFD feeding also increased fasting concentrations of total cholesterol (Fig. 7i) and triglycerides (Fig. 7j). Fasting NEFA concentrations were not affected by diet or genotype (Fig. 7k). Therefore, disrupting skeletal glucocorticoid signaling not only prevented HFD-induced bone loss but also markedly attenuated the development of HFD-induced obesity and its metabolic complications, including insulin resistance, glucose intolerance, and dyslipidemia.

## Discussion

Chronic consumption of HFDs, particularly when rich in saturated fat, promotes bone loss in mice^4–11^ and humans.^12,13^ While diets containing up to 60% of energy intake from fat have been used previously to study bone loss in mice,^7,9–11,14,15^ we used a HFD containing 43% fat (by energy)^4,5,8^ to induce bone loss as well as obesity and its metabolic sequelae (insulin resistance, hyperglycemia and dyslipidemia).^38^ In the present study, the composition of the diet corresponds more closely with the average fat intake in Western countries,^39^ and we used this model to comprehensively investigate the mechanisms contributing to HFD-induced bone loss in mice. Our results highlight a critical role for local osteoblastic/osteocytic glucocorticoid signaling in the deleterious effects of HFD on the skeleton.

Feeding wild-type CD-1 mice a diet of 43% fat for 18 weeks reduced trabecular volume and Ct.Th by 28% and 14%, respectively, compared to chow-fed controls. This degree of bone loss is comparable to previous studies of similar duration, many of which used a diet containing 60% fat.^4,6,7,9–11^ Although there is agreement that HFDs promote bone loss, there is little consensus as to its underlying cause, with studies implicating both reduced bone formation^8,10^ and increased bone resorption.^4–6,10,40^ In our study, HFD-induced bone loss was due to impaired bone formation and occurred without obvious changes in bone resorption. In addition to differences in dietary fat content, the inconsistencies between studies may also reflect variation in the age or strain of the mice studied. Bone loss was not observed after 8 weeks of HFD (Fig. S1).

Our second main finding was that local glucocorticoid signaling in osteoblasts/osteocytes is required, at least in part, for the negative effects of HFD on bone formation. Although it is widely accepted that exogenous glucocorticoids exert deleterious effects on bone, we have previously shown that endogenous glucocorticoid signaling is required for the commitment and osteogenic differentiation of osteoblast precursors.^41^ In the present study, HFD feeding activated glucocorticoid signaling locally in bone, independent of circulating corticosterone concentrations. In HFD-fed mice lacking osteoblastic/osteocytic glucocorticoid signaling (HSD2^OB/OCY^-tg mice), reductions in trabecular bone volume and Ct.Th were only 17% and 6%, respectively, approximately half those observed in HFD-fed wild-type mice with intact glucocorticoid signaling.

Interestingly, bone formation was not reduced by HFD feeding in HSD2^OB/OCY^-tg mice; rather, it was more than doubled compared to chow-fed mice, suggesting that the absence of local glucocorticoid signaling led to a beneficial adaptation to HFD feeding, which involved increased osteoblast activity. This conclusion was supported by increases in skeletal *Col1a1* mRNA expression, skeletal glucose uptake (see below), and serum P1NP concentrations in HFD-fed HSD2^OB/OCY^-tg mice. Of note, HSD2^OB/OCY^-tg mice were also protected against HFD-induced damage to the LCN, which is responsible for orchestrating bone remodeling throughout life. We therefore propose that under normal conditions, induction of skeletal glucocorticoid signaling by a HFD inhibits bone formation and thereby causes bone loss. It follows that disruption of skeletal glucocorticoid signaling enables HSD2^OB/OCY^-tg mice to resist HFD-induced bone loss by increasing bone formation.

This conclusion is supported by the marked, 4.5-fold increase in skeletal glucose uptake in HFD-fed HSD2^OB/OCY^-tg mice, which is symptomatic of increased metabolic activity in osteoblasts and osteocytes. Bone formation is an energy-intensive process, and as they mature, osteoblasts become increasingly dependent on glucose uptake^33^ and aerobic glycolysis^32^ to meet their energetic demands. Studies in mice over a decade ago showed that inhibiting glucose metabolism in osteoblastic cells suppresses bone formation.^28,29^ Conversely, stimulating glycolysis in osteoblasts increases bone formation.^31,42^

Although skeletal glucose uptake (per unit mass) is lower than that in other tissues, such as skeletal muscle and adipose tissue, its sheer size and cellular density (estimate of ~42 billion osteocytes within the average adult human skeleton) suggest that it makes a significant contribution to overall glucose homeostasis. In anesthetized mice, the skeleton accounts for ~15% of total glucose uptake.^42^ The wider metabolic significance of the increased glucose uptake into osteoblastic cells is well illustrated here by the phenotype of HFD-fed HSD2^OB/OCY^-tg mice: compared to wild-type mice, the development of obesity, insulin resistance, glucose intolerance, and dyslipidemia in HFD-fed HSD2^OB/OCY^-tg mice were all markedly attenuated, despite identical caloric intake. Similarly, other studies of mice with increased glucose uptake and utilization in bone also display improved whole-body glucose tolerance and protection against central obesity during aging,^42^ while inhibiting glucose uptake in osteoblasts and osteocytes is accompanied by obesity, glucose intolerance, and insulin resistance in mice.^29,43,44^

Taken together, these findings support the emerging view that glucose metabolism in osteoblasts and osteocytes makes a substantial contribution to energy homeostasis. Thus, HFD-fed HSD2^OB/OCY^-tg mice possess a ‘metabolic sink’ for excess dietary energy intake, which allows them to increase their energy expenditure, preventing weight gain and, at the same time, excessive bone loss. Our data suggest that the high rates of glucose uptake in osteoblasts and osteocytes of HFD-fed HSD2^OB/OCY^-tg mice may prevent obesity and its metabolic sequelae via higher rates of bone formation and maintenance of the LCN, respectively. Future studies will be required to understand the energy requirements of osteocytes and to measure their contribution to energy homeostasis.

We did not obtain evidence to suggest that the beneficial effects of increasing glucose metabolism in osteoblasts and osteocytes are relayed to other tissues via osteoblast-derived molecules. We did not observe any differences in the serum concentrations or skeletal mRNA expression of osteocalcin or lipocalin-2, two osteoblast-derived peptides reported to influence glucose homeostasis^45^ and appetite,^46^ respectively (Fig. S3). The lack of an obvious skeletal-derived endocrine effect has also been reported in many other recent studies.^24,42,47–49^

Instead, our results implicate Wnt signaling as a potential molecular link between increased skeletal glucose uptake and enhanced bone formation in HFD-fed HSD2^OB/OCY^-tg mice. Wnt signaling is a major driver of bone formation,^27^ determining nearly all aspects of osteoblast function,^50^ and is inhibited by glucocorticoids.^51,52^ In mice, long-term treatment with exogenous glucocorticoids (prednisolone) increases skeletal *Sost*/sclerostin expression,^53^ and several glucocorticoid response elements have been identified in the *SOST* promoter.^54^ Consistent with previous studies,^55,56^ we found that HFD feeding induced both glucocorticoid signaling in bone and the expression of *Sost*/sclerostin. In HSD2^OB/OCY^-tg mice, however, induction of sclerostin by HFD was attenuated, more osteoblasts and osteocytes expressed β-catenin, and the expression levels of the downstream Wnt signaling molecules *Tcf7*, *Tcf7l2*, and *Bmp4* were all increased, suggesting that Wnt signaling, and therefore bone formation, was instead stimulated in these mice. Interestingly, Wnt signaling stimulates aerobic glycolysis in osteoblastic precursors and promotes their differentiation.^30,35^ Our results therefore suggest that glucocorticoid signaling in osteoblasts and osteocytes is necessary for the inhibition of Wnt signaling by HFD.

In the present study, only male mice developed insulin resistance in response to HFD. Sexual dimorphism is frequently reported in murine models of HFD-induced dysmetabolism.^57^ We have recently shown a permissive role for androgens in glucocorticoid-induced insulin resistance.^58^ Last, it is possible that altered secretion of adipose-derived signaling molecules (such as leptin) from expanded fat stores could have influenced bone formation in our model.^59^

In summary, we implicated suppression of bone formation as the major mechanism underlying HFD-induced bone loss and identified a critical role for osteoblastic/osteocytic glucocorticoid signaling in this process. Interestingly, intracellular glucocorticoid signaling (and therefore glucose utilization) in osteoblasts and osteocytes was also an important determinant of the whole-body metabolic adaptation to chronic HFD feeding. Our findings suggest that the skeleton may be at least partially responsible for the beneficial metabolic effects of 11β-HSD1 inhibitors, which have been shown to improve components of metabolic syndrome in both mice^60^ and humans.^61^ This study therefore highlights osteoblastic cells as a key site of intervention not only for glucocorticoid-induced osteoporosis but also for the treatment of obesity and its associated metabolic consequences.

## Materials and methods

### Study design, primary outcome variables, and power calculations

This study was designed to investigate the skeletal response to 18 weeks of chow or HFD feeding in male CD-1 mice with either intact or disrupted skeletal glucocorticoid signaling. Based on previous studies of HFD-induced bone loss (12–16 weeks duration),^4,7,9–11^ we expected a 20%–30% loss of trabecular bone volume (BV/TV) in our 18-week study. We assumed a mean trabecular BV/TV of 13% ± 3% (SD) in chow-fed wild-type mice; therefore, a sample size of 11 mice per group would provide us with 62%–92% power to detect a HFD-induced decrease of 20%–30% at a two-sided *α* = 0.05.

### Mouse model, housing, and HFD feeding

Wild-type and HSD2^OB/OCY^-tg mice were housed at the ANZAC Research Institute at 24 °C with a 12-h light/12-h dark cycle. Water and food were provided ad libitum. Two cohorts of 7-week-old wild-type and transgenic mice were studied, with mice fed either regular rodent chow (#SF14-008, 14.3% energy from fat) or HFD (#SF14-144, 43.0% energy from fat, both from Specialty Feeds, Glen Forrest, WA, Australia) for 8 or 18 weeks. Food intake was measured at 4, 8, and 16 weeks, and body composition was determined using a Lunar PIXImus instrument (GE Healthcare, Parramatta, NSW, Australia).

At the end of the study, blood was collected from anesthetized mice by cardiac puncture. Tissues were dissected out, immersed in liquid N_2_, and transferred to a −80 °C freezer. For bone, the femur was collected, the heads were removed, and the remaining shaft was flushed of bone marrow using sterile PBS before snap freezing.

### Micro-computed tomography (micro-CT)

A Skyscan 1172 instrument (Bruker MicroCT, Kontich, Belgium) was used to perform micro-CT analysis of the L3 vertebrae and tibiae, according to the manufacturer’s instructions (see ref. ^26^). Scanning was performed at 100 keV, 167 μA, and 1 475 ms without filter. In total, each sample yielded ~1 100 projections at 7.6 μm per pixel resolution. NRecon and CTAn software (Bruker MicroCT) were used for data analysis, and the region of interest was selected automatically. The entire space between the cranial and caudal growth plates of L3 was used to obtain the vertebral trabecular parameters. Morphological measurements included the bone volume fraction (BV/TV), Tb.N., Tb.Th., and trabecular separation for trabecular bone and the cortical thickness (Ct.Th), cortical volume, Ct.Ar, and cortical area fraction (Ct.Ar/Tt.Ar) for cortical bone.

### Dynamic histomorphometry

Mice were injected with calcein (30 mg·kg^−1^ i.p., Sigma-Aldrich, Castle Hill, NSW, Australia) 10 and 3 days prior to sacrifice. Following dissection, methylmethacrylate resin was used for embedding tibiae (as described in ref. ^62^), and tissues were cut into 5-μm sections and stained with xylenol orange. The proximal tibia was analyzed using Osteomeasure software (Osteometrics, Decatur, GA, U.S.A.). The MAR and BFR in the trabeculae were calculated as described in ref. ^19^

### Histomorphometry

Following micro-CT analysis, fixed bones were decalcified in 10% EDTA, pH 7.5 (Merck, Kenilworth, NJ, U.S.A.), and paraffin-embedded. Serial 5-μm sections were stained with hematoxylin and eosin. To identify osteoclasts, sections were stained for tartrate-resistant acid phosphatase (TRAP) using naphthol-AS-BI-phosphate as a substrate and fast red violet LB salt as a detection agent (both from Sigma-Aldrich). To analyze the trabecular bone, a standardized region of interest was placed in the axial middle of the tibia, 0.5 mm below the growth plate. For cortical bone, a region of interest covering the area 2.5 mm–3.5 mm below the growth plate was assessed. TRAP-positive cells were counted to determine the osteoclast number and surface relative to the bone surface. Osteoblasts were identified by morphology. All measurements were performed at ×200 magnification.

### Immunohistochemistry

After fixation in 4% paraformaldehyde for 48 h at 4 °C, tissues were embedded in paraffin and cut into 5-μm sections. Antigen retrieval was performed using 1-mmol·L^−1^ citrate buffer or 1-mmol·L^−1^ EDTA buffer at 65 °C for 60 min. After blocking endogenous peroxidases, the sections were incubated overnight with the primary antibody (rabbit anti-rat DMP1 antibody, #LS-B11226, LSBio, Seattle, WA, U.S.A., at a 1:200 dilution or rabbit anti-mouse β-catenin antibody #9562, Cell Signaling Technology, Danvers, MA, U.S.A., at a 1:50 dilution). Subsequently, sections were incubated with a biotinylated anti-rabbit secondary antibody (1:200 dilution, Vector Laboratories, Burlingame CA, U.S.A.). This was followed by incubation with avidin–biotin–peroxidase (Vector Laboratories). Visualization was performed using diaminobenzidine (DAKO, Carpinteria, CA, U.S.A.). The sections were then counterstained with hematoxylin. Staining was quantified using ImageJ software (National Institutes of Health, Bethesda, MD, U.S.A.), and positive cells were identified using Fiji software (https://fiji.sc). Investigators were blinded to diet and genotype.

### Silver nitrate staining

Silver nitrate staining was used to identify lacunae in sagittal sections of long bones from wild-type and HSD2^OB/OCY^-tg mice. Briefly, sections were heated to 63 °C for 10 min and rehydrated in 100%, 90%, and 70% ethanol before being incubated in fresh solution containing silver nitrate (33% v/v, in water), gelatin (0.7% w/v), and formic acid (0.4% v/v) for 1 h in the dark. Sections were then incubated in 5% sodium thiosulfate (w/v) for 5 min, followed by dehydration in ethanol and coverslipping. Lacunar and perilacunar areas were determined using ImageJ by an investigator blinded to diet and genotype.

### Glucose homeostasis and tissue-specific glucose uptake

Tolerance tests for insulin and glucose (ITTs and GTTs, respectively) were performed at the timepoints stated after a 6-h fast. ITTs were performed by intraperitoneal injection of 0.75-U·kg^−1^ insulin (Humalog, Lilly, Indianapolis, IN, U.S.A.). GTTs were performed using a dose of 1.5-g·kg^−1^ glucose delivered by oral gavage. In both cases, blood samples were collected via the tail vein, and an Accu-check glucometer (Roche, North Ryde, NSW, Australia) was used to determine blood glucose concentrations. Glucose uptake into tissues was determined using a radiolabeled glucose tolerance test (glucose, 2 g·kg^−1^ i.p.), as described in ref. ^63^

### Indirect calorimetry

Energy expenditure was measured using a Promethion M indirect calorimetry system using MetaScreen v. 1.6.2 software (Sable Systems, Las Vegas, NV, U.S.A.). After 48 h of acclimation, energy expenditure was measured for 72 h, and mice had free access to food and water. A flow rate of 2 000 mL·min^−1^ was used. O_2_ consumption and CO_2_ production were measured for each mouse at 5-min intervals. The Weir equation (kcal per h = 60*(0.003 941*VO_2_ + 0.001 106*VCO_2_) was used to calculate energy expenditure, and the results were normalized to lean body mass. Raw data were processed using ExpeData v.1.4.3 (Sable Systems).

### Gene expression

Total RNA and protein were extracted from tissues using TRIzol reagent (Sigma-Aldrich), followed by an on-column clean-up (Nucleospin, Macherey-Nagel, Düren, Germany). cDNA was synthesized using Superscript III First-Strand Synthesis Supermix (Invitrogen, Carlsbad, CA, U.S.A.). Real-time PCR was performed using SsoAdvanced Universal SYBR Green Supermix on a CFX Connect Real-Time PCR Detection system (both from Bio-Rad, Hercules, CA, U.S.A.). Gene expression was normalized to *18S* expression, and the 2^−ΔΔCt^ method was used to calculate relative expression. Primer sequences for *Hsd11b1*, *Tsc22d3*, *Col1a1*, *Sost*, *Tcf7*, *Tcf7l2*, *Bmp4, Glut1, Glut4, Ocn*, and *Lcn* are provided in Table S1.

### Immunoblotting

Protein was extracted from tissues using TRIzol reagent (Sigma-Aldrich) according to the manufacturer’s protocol. Protein was precipitated with isopropanol and resuspended in solubilization buffer (100-mmol·L^−1^ Tris·HCl, 140-mmol·L^−1^ NaCl, 20-mmol·L^−1^ EDTA, 5% SDS and protease/phosphatase inhibitors). Twenty micrograms of protein were electrophoresed in a 4%–12% Tris-Bis acrylamide gel with MES buffer (both from Invitrogen). Electrophoresed proteins were transferred to PVDF membranes using an iBlot2 instrument (Thermo Fisher, North Ryde, NSW, Australia) and blocked before being probed with primary antibodies directed against sclerostin (0.2 μg·mL^−1^, #AF1589, R&D Systems, Minneapolis, MN, U.S.A.) or beta-actin (1:1 000, #4697, Cell Signaling Technology, Danvers, MA, U.S.A.). After incubation with appropriate secondary antibodies, bands were visualized using the chemiluminescent substrate ECL Plus (Thermo Fisher) and a ChemiDoc XRS+ instrument using Image Lab software (Bio-Rad).

### Biochemical measurements

The concentrations of triglycerides, NEFAs, and cholesterol were measured in freshly thawed samples of fasted serum using colorimetric enzymatic kits from WAKO Diagnostics (Osaka, Japan). Leptin concentrations were measured using a suspension bead array immunoassay (Bio-Plex Pro Mouse Diabetes 8-Plex) on a Bio-Plex series 100 instrument (both from Bio-Rad). Commercially available ELISAs were used for the measurement of serum insulin (Mercodia, Uppsala, Sweden), lipocalin-2 (R&D Systems, Minneapolis, MN, United States), and osteocalcin (Takara Bio, Mountain View, CA, U.S.A.). Serum concentrations of mouse tartrate-resistant acid phosphatase 5b (TRAP5b) and PINP were quantified using assays from Immunodiagnostic Services (Bolden, Tyne & Wear, U.K.). Liquid chromatography–tandem mass spectrometry was used to measure serum corticosterone levels.^64^

### Statistical analysis

All statistical calculations were performed using Prism software (version 8.3.0, GraphPad, San Diego, CA, U.S.A.). The Kolmogorov–Smirnov test was used to assess the distribution of continuous variables. The effects of diet (chow, HFD) and genotype (wild-type, HSD2^OB/OCY^-tg) and potential diet × genotype interactions were tested by two-way ANOVA. Tukey’s multiple comparison test was used for post hoc testing. Insulin and glucose tolerance test results were summarized using the area under the curve, calculated with a trapezoidal method in Microsoft Excel. Blood glucose concentrations during the ITT were expressed as a percentage of basal levels. Differences in the distribution of LCN components were assessed using a *χ*^2^ test. In all cases, a two-tailed *P* value < 0.05 was statistically significant.

## Supplementary information

Supplementary Information

## Data Availability

The data that support the findings of the study are available from the corresponding author upon reasonable request.
