# Supplementary material for: Osteoblastic glucocorticoid signaling exacerbates high-fat-diet- induced bone loss and obesity
Source: Bone Res. 2021 Sep 1;9:40. doi: 10.1038/s41413-021-00159-9 (PMC8408138; doi:10.1038/s41413-021-00159-9)
Supplement: Supplementary file 1 — Supplementary Information [file 41413_2021_159_MOESM1_ESM.docx]

**Supplementary Information for Kim S *et al*. “Osteoblastic Glucocorticoid Signalling Exacerbates High-Fat Diet-Induced Bone Loss and Obesity”.**

**Submitted to *Bone Research*.**

**Table S1. Sequences for PCR primers**

| ***Gene product*** | ***Forward primer (5’ to 3’)*** | ***Reverse primer (5’ to 3’)*** |
| --- | --- | --- |
| *18S* | CATGATTAAGAGGGACGGC | TTCAGCTTTGCAACCATACTC |
| *Bmp4* | TCAGAATCAGCCGATCGTT | GCAGTAGAAGGCCTGGTAGC |
| *Col1a1* | CCTGCCTGCTTCGTGTAAA | ATTGAGTTTGGGTTGTTCGTCT |
| *Gilz* | CTCGTGAAGAACCACCTGATG | ACTTACACCGCAGAACCACC |
| *Glut1* | TGTGGCTGCTGTGCTTATG | CTGGGATGAAGATGACACTGAG |
| *Glut4* | CTGCCTGAGATGGAGAAACTACT | CCTCGGAGATGACCTTGAAG |
| *Hsd11b1* | TCCCTGTTTGATGGCAGTTA | TGGAGCATTTCTGGTCTGAA |
| *Lcn2* | ATTACCCTGTATGGAAGAACCAA | AAGATGATGTTGTCGTCCTTGA |
| *Ocn* | GCTCTGTCTCTCTGACCTCACA | TAGATGCGTTTGTAGGCGG |
| *Sost* | AGGAGAGAGAGCGTTTGTAACA | GCTTTCAGTCTTTGTGGATGAGT |
| *Tcf7* | ACCCTCCAGAATCCACAGATAC | ATGTTGCCTCCTCCTGAGTTAG |
| *Tcf7l2* | GGAACTAGTCTTCTCTCAGCCAA | CGTCATCGGATTTGATCTCA |

**
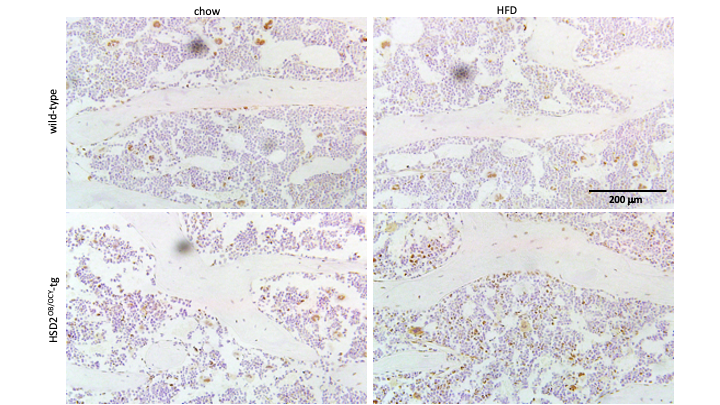
**

**Figure S2. β-catenin staining of sections of trabecular bone from wild-type and HSD2^OB/OCY^-tg mice fed either chow or HFD for 18 weeks.**

**
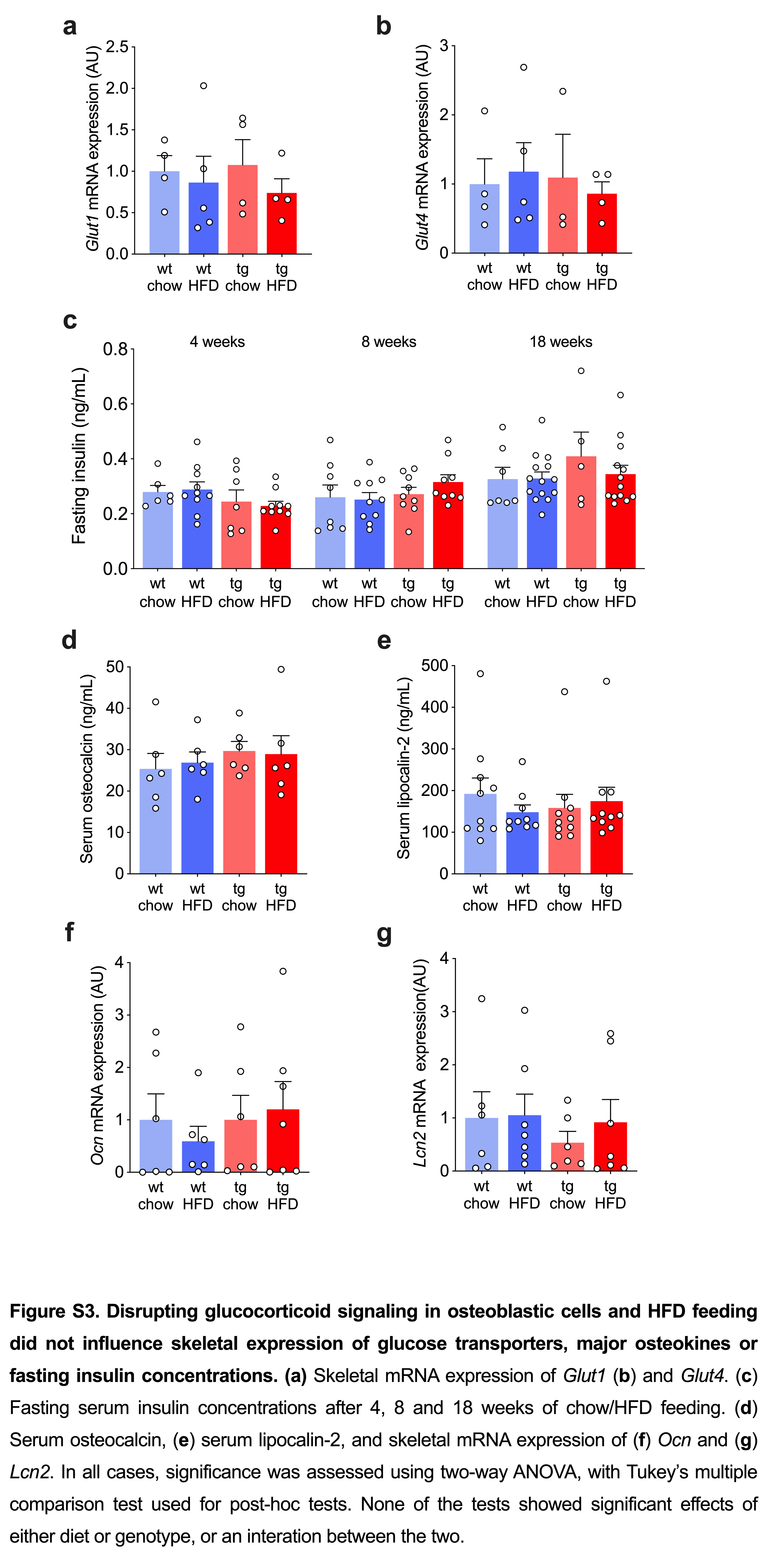
**
